# How can engagement of adolescents in antenatal care be enhanced? Learning from the perspectives of young mothers in Ghana and Tanzania

**DOI:** 10.1186/s12884-019-2326-3

**Published:** 2019-05-23

**Authors:** Kristy Hackett, Lindsey Lenters, Ashley Vandermorris, Curtis LaFleur, Sam Newton, Sidney Ndeki, Stanley Zlotkin

**Affiliations:** 1 Department of Global Health and Population, Harvard T. H. Chan School of Public Health, 677 Huntington Ave, Boston, MA 02115 USA; 2 Dalla Lana School of Public Health, University of Toronto, 155 College St, Toronto, ON M5T 3M7 Canada; 3 Lawrence S. Bloomberg Faculty of Nursing, University of Toronto, 155 College St, Toronto, ON M5T 1P8 Canada; 4 Centre for Global Child Health, Hospital for Sick Children, 686 Bay Street, 11th Floor, Toronto, ON M5G 0A4 Canada; 5 Ontario Shores Centre for Mental Health Sciences, 700 Gordon St. W, Whitby, ON L1N 5S9 Canada; 6 Kwame Nkrumah University of Science and Technology, Accra Road, Kumasi, Ghana; 7 PRAXIS Tanzania, 5th Floor Mariam Tower, Ilala, Shaurimoyo Street, Dar es salaam, Tanzania

**Keywords:** Adolescents, Health seeking, Behaviour change, Antenatal care, Sub-Saharan Africa, Qualitative research, Pregnancy, Childbirth, Health services

## Abstract

**Background:**

Adolescents are especially vulnerable due to increased biological, social and economic risks associated with early pregnancy and childbirth, yet most pregnancy and childbirth-related complications are preventable through a combination of proven, cost-effective clinical interventions including timely antenatal care (ANC). The voices and specific needs of adolescents are currently underrepresented in the literature on antenatal and maternity care. Objectives were to a) increase our understanding of adolescents’ experiences with, and perceptions of, ANC and b) explore how these perspectives might be applied towards future initiatives to enhance adolescent care-seeking behaviour.

**Methods:**

This cross-sectional qualitative study employed 14 focus group discussions with 112 adolescents aged 15–20 years in Singida Region in Tanzania and Volta and Eastern Regions in Ghana who had accessed ANC during their most recent pregnancy. We were particularly interested in what these young women valued and understood about their ANC experience, as this would provide insights into what factors motivated them to seek care. Transcripts were analyzed using conventional content analysis. Based on emergent themes and drawing on the Health Belief Model (HBM) as an analytical tool, a conceptual framework was developed to illustrate the myriad factors influencing adolescents’ decision to attend ANC.

**Results:**

Interpreting results through an adapted HBM demonstrates that adolescent health-seeking behaviour can vary widely among individuals and within communities, is shaped by the opinions of family members and peers, and is intrinsically influenced by broader health systems-level factors.

**Conclusions:**

The results led to our development of an adapted theory-based framework to illustrate the complexity of adolescent care-seeking during pregnancy in resource-poor settings. We demonstrate that while an adolescent mother is capable of exercising her own agency, she is also developmentally vulnerable to external influences and must be supported in her ability to make autonomous decisions. While the model presented here focuses specifically on ANC utilization, it may have applications for understanding how adolescents engage with health services more broadly.

**Electronic supplementary material:**

The online version of this article (10.1186/s12884-019-2326-3) contains supplementary material, which is available to authorized users.

## Background

Adolescents are especially vulnerable due to increased biological, social and economic risks associated with early pregnancy and childbirth. While adolescent pregnancy rates have generally decreased over the past few decades, these decreases have not been uniform. Worldwide about 16 million girls aged 15–19 years give birth annually, and maternal conditions such as hemorrhage, sepsis, hypertensive disorders, obstructed labour, and complications of abortion remain the leading cause of death among adolescent girls 15–19 years old [1]. A majority of these births occur in low- and middle-income countries (LMIC), and within these countries there is uneven distribution among socio-economic groups [2]. Poor maternal health outcomes for both adult women and adolescent girls in LMIC result from numerous underlying causes, yet most pregnancy and childbirth-related complications are preventable through a combination of proven, cost-effective clinical interventions [3]. Timely antenatal care (ANC) facilitates contact with the health system, and thus provides an opportunity to screen for, prevent or treat such complications.

ANC is defined as “care provided by skilled healthcare professionals to pregnant women and adolescent girls in order to ensure the best health conditions for both mother and baby during pregnancy” and includes four categories of interventions: 1) risk identification; 2) prevention; 3) management of pregnancy-related or concurrent diseases; and 4) health promotion and education, including counseling on the importance of giving birth with assistance from a skilled provider [4]. ANC is a critical window of opportunity because it often serves as an entry point into the formal health system, enabling access to health services for future maternal and child health needs [5]. ANC visits also provide opportunities for identification and management of obstetric complications, testing for human immunodeficiency virus (HIV) and other sexually transmitted infections (STI), malaria prevention during pregnancy, and administration of tetanus toxoid vaccines [4].

Timely uptake of ANC tends to be lower among adolescents compared to older women due to their limited autonomy with respect to decision-making and financial resources, among other factors. Adolescent females face greater barriers to accessing ANC, increasing the likelihood of complications related to pregnancy and childbirth [6]. They are also more likely to be stigmatized particularly when pregnancy occurs outside marriage. As a result, they may have lower levels of family and social support as compared to older women [7, 8]. In some settings, adolescents may delay pregnancy disclosure out of fear of expulsion from school [9]. Health system-level factors such as lack of age appropriate services and negative attitudes of healthcare providers may also discourage adolescents from seeking ANC [10, 11].

The most recent World Health Organization (WHO) guidelines recommend a shift towards women-centred ANC, emphasizing a “positive pregnancy experience.” [4]. At present, many women in LMIC choose not to, or are unable to access recommended ANC due to a host of individual, socio-cultural, and health systems-level factors. While a large body of research has documented barriers and facilitators of ANC utilization among women in many LMIC, there is little evidence on how current ANC delivery models address the specific needs of adolescent girls. Despite global calls for more “adolescent friendly” health facilities and greater adolescent participation in shaping health services, the voices of adolescents themselves are currently underrepresented [1]. A deeper understanding of adolescents’ experiences with health systems is needed to inform more effective policy and program implementation.

This project was part of a larger study designed to help fill this gap by exploring the perceptions, care seeking behaviour, and experiences related to ANC among adolescent mothers in Tanzania and Ghana, two countries where rates of adolescent pregnancy are unacceptably high. In Tanzania, the proportion of women aged 15–19 who have begun childbearing or are currently pregnant increased from 23% in 2010 to 27% in 2016 [12]. In Ghana, this indicator increased slightly from 13% in 2008 to 14% in 2014 [13]. The objectives of this study were to a) increase our understanding of adolescents’ experiences with, and perceptions of, ANC and b) explore how these perspectives might be applied towards future initiatives to enhance adolescent care-seeking behaviour.

## Methods

### Study design

This cross sectional qualitative study involved focus group discussions (FGD) with primiparous adolescents who had accessed ANC while pregnant with their first child. We were particularly interested in what these young women valued and understood about their ANC experience, as this would provide insights into what factors enabled them to seek care. Data collection occurred within the context of existing community-based programs implemented by two large international non-governmental organizations (NGOs): Plan International (in Ghana) and World Vision (in Tanzania).

### Site selection and sampling

Data was collected from within specific program implementation areas in Volta and Eastern regions of Ghana and the Singida region of Tanzania. The research team had no role in the selection of country sites; rather, the implementing NGOs made this decision based on a combination of maternal, newborn, child, and adolescent health indicators targeted by their programs.

In each country, a two-staged sampling strategy was employed. First, we purposively selected two health centres from the two NGO programmatic areas in each country to capture heterogeneity of experience and programmatic context (total facilities = 8). While not a representative sample, we anticipated that collecting data in four facilities in each country would provide a reasonable snapshot of the situation in each context, and a manageable amount of data given time and resource constraints. Since the aim was to understand the experiences of adolescents who *had* accessed ANC, we intentionally selected high functioning health facilities that met the following criteria: 1) had an active and functioning community health worker team (Tanzania) or community mobilizer team (Ghana) who could assist the research team with locating potential study participants; 2) were geographically accessible to the field teams; 3) had high ANC attendance to ensure a sufficient number of eligible participants; and 4) offered comparable packages of maternal health services (to allow for comparison across sites).

The second sampling stage involved a random selection of adolescents from ANC rosters in each health centre. Eligible participants were screened for the following inclusion criteria: 1) primiparous female aged 15–19 years at the time of their ANC visit; 2) gave birth within the past 12 months (live birth or stillbirth); 3) attended ANC at a target health centre at least once during this pregnancy; and 4) resided in the catchment area of a target health centre. Screening was based on data obtained from health facility ANC records. Six to eight participants were randomly selected and invited to participate in a FGD by a community health worker or community mobilizer. In some cases, we found on the day of the FGD that selected participants had actually given birth more than 12 months prior (maximum 21 months), which may have been due to inaccurate facility records. This was true for five participants in Tanzania, and one participant in Ghana. Since these individuals had travelled to the interview site and made arrangements to participate, they were included in the study.

### FGD instrument

The consent documents and FGD guide were developed in English and translated into the local languages of each country/region (Swahili in Tanzania, and Twi, Ewe or Krobo in Ghana). All members of the local research teams were proficient in these languages. Both instruments were pre-tested prior to use, and language was adapted to each context to ensure phrasing was culturally appropriate and easily interpreted by study participants.

We developed a semi-structured FGD guide that covered a range of pre-determined topics related to the study aims (Additional file 1). The guide consisted of open-ended questions and facilitator probes to encourage elaboration by participants where appropriate. The guide also included several hypothetical case examples, which the facilitator read aloud to participants. The facilitator then asked participants to offer their opinions of the situation. This technique was particularly useful for more sensitive issues, as participants could share their ideas freely without relating opinions directly to personal experiences.

### Data collection

In both Ghana and Tanzania, a local FGD facilitator and research assistants (all females) were recruited and trained by the Canadian research team. To ensure a comfortable and familiar environment conducive to open discussion, community health workers/mobilizers identified “neutral” spaces (i.e. far from health facilities and ANC providers) that were easily accessible to participants. Village-based indoor spaces were chosen to protect the privacy of the participants and to facilitate digital recording of the conversations. Facilitators were trained to guide discussion in a flexible manner, allowing participants to steer the conversation towards relevant topics of interest. They were also trained to ask follow-up questions and use appropriate probing techniques to encourage rich explanations, and to avoid prompting or asking leading questions [14]. Note-takers took detailed written notes to document observations of group interactions, facial expressions, and other non-verbal behaviours to provide additional context to the analysis.

### Data management and analysis

All digital FGD recordings were transcribed and translated from Swahili, Twi, Ewe and Krobo to English by the in-country FGD facilitators. Transcription and translation of the audio recordings occurred simultaneously. Participant conversations were transcribed verbatim without condensing or summarizing responses, and notes of any important context were embedded within the transcripts. One bilingual member of each in-country research team conducted quality and consistency checks of each transcript using a standard quality assurance protocol and assessment form (see Additional file 2). This stage was critical to ensure the accuracy of translated transcripts from the local language into English and reduce the chances of accidental errors, misinterpretations, or omissions.

The analysis team held debriefing meetings with facilitators shortly after each FGD to discuss each session’s content. This provided an opportunity to discuss any challenges that emerged during FGDs, to identify emergent themes in the data, and to discuss important contextual information that would help frame the analysis and interpretation. This iterative approach served as a critical first step in the data analysis process.

Data analysis was guided by the principles of conventional content analysis [15]. The aims of this analysis were twofold: first, to describe the phenomenon under study (adolescent girls’ experiences and decision-making processes with respect to ANC), and secondly to draw linkages to existing theories and/or conceptual models by comparing and contrasting results with similar studies. Researchers adopted an inductive approach, whereby themes and patterns emerge from the data, rather than a deductive approach where data analysis aims to test the validity of specific pre-existing ideas and theories [16]. An initial coding framework was developed using QSR International’s NVivo 10 software [17] based on broad themes identified through an initial review of the transcripts.

Two experienced qualitative researchers coded each transcript independently. The ‘primary coder’ analyzed the transcripts and made minor changes to the coding framework as new themes emerged from the data. A ‘secondary coder’ then reviewed the primary coder’s analysis and made additions or changes based on their impression of the data. Each coder kept a ‘coding diary’ within NVivo to document ideas about emerging themes, surprising findings, and other details useful for analyzing and interpreting the data. Regular meetings were held to discuss and resolve any discrepancies. These discussions guided interpretation of emergent findings, and enabled the team to draw connections within and across FGDs.

Once coding was finalized, the research team independently ran queries, read and re-read the coded transcripts to synthesize themes and to identify interesting outliers and surprising features of the data. The analysis team then summarized findings and questions in briefing notes that were consolidated and circulated to all research team members and FGD facilitators. Group calls were held to discuss all parties’ impressions of the findings, to validate interpretations of the data, and to fill in any gaps in the analysis. This process was designed to enhance the internal validity of the analysis [15].

### The health belief model as an analytical tool

**Fig. 1:**
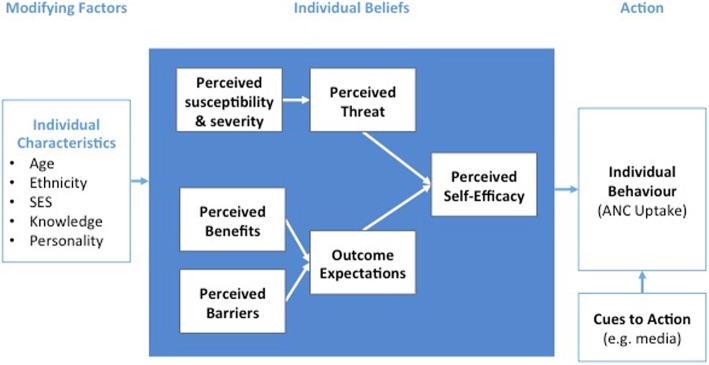
Health Belief Model, adapted from Nutbeam & Harris 1998; Glanz et al., 2008

The Health Belief Model (HBM) was selected as the principal framework to guide analysis. Developed in the 1950s, the HBM is one of the most commonly used frameworks for understanding individual health behaviours [18, 19]. The HBM (Fig. 1) includes a number of core constructs that help to explain why individuals take action to prevent or control illness/health conditions, including: one’s beliefs about susceptibility to a condition and severity of that condition, considerations of the benefits and barriers to a health-promoting behaviour, cues to action, and self-efficacy [19]. While the HBM model served as a useful starting point for framing emergent study findings, we learned during early stages of analysis that it was not a perfect fit for the data. Given the multitude of factors affecting ANC utilization that are beyond control of individual adolescents, we adapted the HBM to incorporate additional constructs from The Theory of Planned Behaviour [20] and ecological models of behaviour change such as Social Cognitive Theory [21]. This adapted model was used as a guide to organize emergent findings from FGD transcripts.

## Results

Eight FGDs were conducted in Tanzania with a total of 62 participants, and six FGDs were conducted in Ghana with a total of 50 participants. Demographic characteristics of FGD participants are presented in Tables 1 and 2.

**Table 1:** Self-reported participant characteristics at the time of data collection, by country

	Ghana [mean (SD), range]	Tanzania [mean (SD), range]
Age of participants (years)	17.8 (1.1), 15–19	18.5 (1.6), 15–20
Age of child (months)	6.6 (1.1), 0.5–19	8.0 (4.9), 1–21
Number of ANC Visits	5.3 (2.1), 1–9	4.2 (1.0), 1–6
Month of gestation at first ANC visit	3.5 (1.6), 1–8	5.0 (1.4), 1–8

**Table 2:** Participant characteristics by country, continued

		Ghana [n (%)]	Tanzania [n (%)]
Relationship Status	Married	9 (18)	36 (58)
	Cohabitating	16 (32)	7 (11)
	Single	25 (50)	19 (30)
Education Level	Primary Only	11 (22)	43 (69)
	Secondary	38 (76)	15 (24)
	No school	1 (2)	4 (7)
Currently in school?	Yes	15 (30)	0 (0)
	No	35 (70)	62 (100)

While geographically and culturally distinct, in general participants from the two research sites held remarkably similar perceptions of ANC, with some key differences.

### Individual beliefs

Across all FGDs, participants expressed a shared perspective that they were not ‘meant’ to be pregnant yet, and that adolescent women faced a higher risk of obstetric complications compared to older women, which put the fetus at risk of adverse birth outcomes. Perceptions of increased obstetrical risks associated with their ‘abnormal’ situation are captured in the model as “perceived susceptibility and severity”. These notions of risk inform the perceived threat associated with being pregnant at a young age. The HBM postulates that the higher perceived susceptibility and severity, the more likely the associated health-promoting behaviour will be practiced. Participants clearly identified ANC as the best practice for mitigating risks associated with pregnancy.

According to the model, when perceived benefits of attending ANC are thought to outweigh perceived barriers, the likelihood that the target behaviour will be performed increases. In both countries, participants generally understood the benefits of ANC, however, there was variability in participants’ expression of enthusiasm for the benefits. In general, Ghanaian adolescents were more vocal about the benefits of ANC, while Tanzanian participants were more subdued in their responses. Perceived barriers to ANC uptake could be categorized into two categories in both countries: tangible costs, such as cost of transport to the health facility, and psychosocial costs, such as fear of discrimination by peers (Table 3).

**Table 3:** Individual beliefs influencing ANC uptake among adolescents

Construct	Definition and application to ANC uptake by adolescents	Illustrative Quote(s)
Perceived Susceptibility	One’s opinion about how vulnerable they are to a condition and its consequences (i.e. that adolescent women are more susceptible to obstetric complications than older women). Individual is more likely to attend ANC if she believes the risk of experiencing illness or pregnancy/obstetric complications as a first time mother is high.	*“She has to go so that she will be taken care of. Some of them [adolescent girls] they don’t have a strong waist [developed pelvis] to go through childbirth.” [GHA-6, R6]*
PerceivedSeverity	One’s opinion of the seriousness of a condition and its consequences. Individual is more likely to attend ANC if she believes that complications or illness during pregnancy, the risk of maternal and/or fetal illness or death is higher among adolescents.	*“I was advised by different people, my friend, but also I thought because nowadays babies are born with disabilities, I better hurry to the clinic.”* * [TZA-3, R4]*
Perceived Benefits	ANC uptake is more likely if one believes early and frequent ANC attendance will minimize risk and severity of illness/ complications. Key benefits of ANC described by participants: - Confirm pregnancy and ensure partner “takes responsibility” - Blood test (HIV) - To receive drugs if necessary - To protect the welfare of the baby	*“We go early because if we delay our boyfriend might deny the pregnancy… If you get impregnated by someone and the person refuses responsibility, a paternity test could be performed to know the real person responsible for the pregnancy. Otherwise you will be left with the responsibility and care of the pregnancy alone.”* *[GHA-6, R3]* *“When you go there your blood is tested to know if you have any form of disease and you would then be given medication for any disease found in your blood.”* *[GHA-6, R1]* *“We feel much healthier and stronger than if we do not go. I was given medicine at ANC.”* *[GHA-4, R1]*
Perceived Barriers	One’s opinion of the tangible and psychological costs of the advised action. ANC uptake is more likely if perceived barriers are lower than perceived benefits. 1) Psychosocial costs: - Shyness, embarrassment - Fear of harsh treatment by nurses - Fear of social discrimination	*“I was scared of getting a full body check-up as I never had one before and I heard that they will completely undress me to be touched.” [TZA-3, R6]* *“For instance you might be a student and when your classmates see you going they could make fun of you.” [GHA-2, R2]*
	2) Tangible costs: - Long distance to walk - Long wait times - Cost of transport - Confirmation of pregnancy (alerting others to status) - Dislike for medications or services - Having to leave school	*“The distance from villages to the facility. We have to walk for a very long distance to reach the clinic and we don’t have transport.” [TZA-5, R1]* *“Some of the medicines smell a lot and I don’t like that medicine” [GHA-4, R1]*
Cues to Action	Strategies to activate “readiness”. ANC uptake is more likely if she receives reminders or public health messages promoting the use of ANC (media, campaigns, school, key social referents etc.) 1. Radio 2. Traditional birth attendant, or community health worker/mobilizer 3. mHealth notifications	*“I receive text messages at times on the dos and don’ts of a pregnant woman. We also get phone calls at times.” [GHA-3, R4]*

The motivation to confirm one’s pregnancy was described as a pivotal determinant of whether, and at what point, adolescents sought ANC. In Ghana, having a professional confirm a pregnancy was seen as a means of ensuring that pregnancy could not be denied by the ‘responsible’ male. Participants who sought early pregnancy confirmation at a health facility could then learn about and enroll in ANC at an earlier gestational age. On the other hand, participants also suggested that associated pregnancy confirmation might prevent or delay ANC uptake because of perceived potential consequences including being forced to leave school (in Tanzania), not being allowed to continue living in the family home (both countries), or fear of possible serious ramifications if they opted for pregnancy termination (as expressed in Ghana):

*“If I want to abort the baby and I go for the session and later abort it maybe the midwife might notice me later and question me and if I tell her the truth she might call the police to arrest me.” [GHA-6, R3]*

**Fig. 2:**
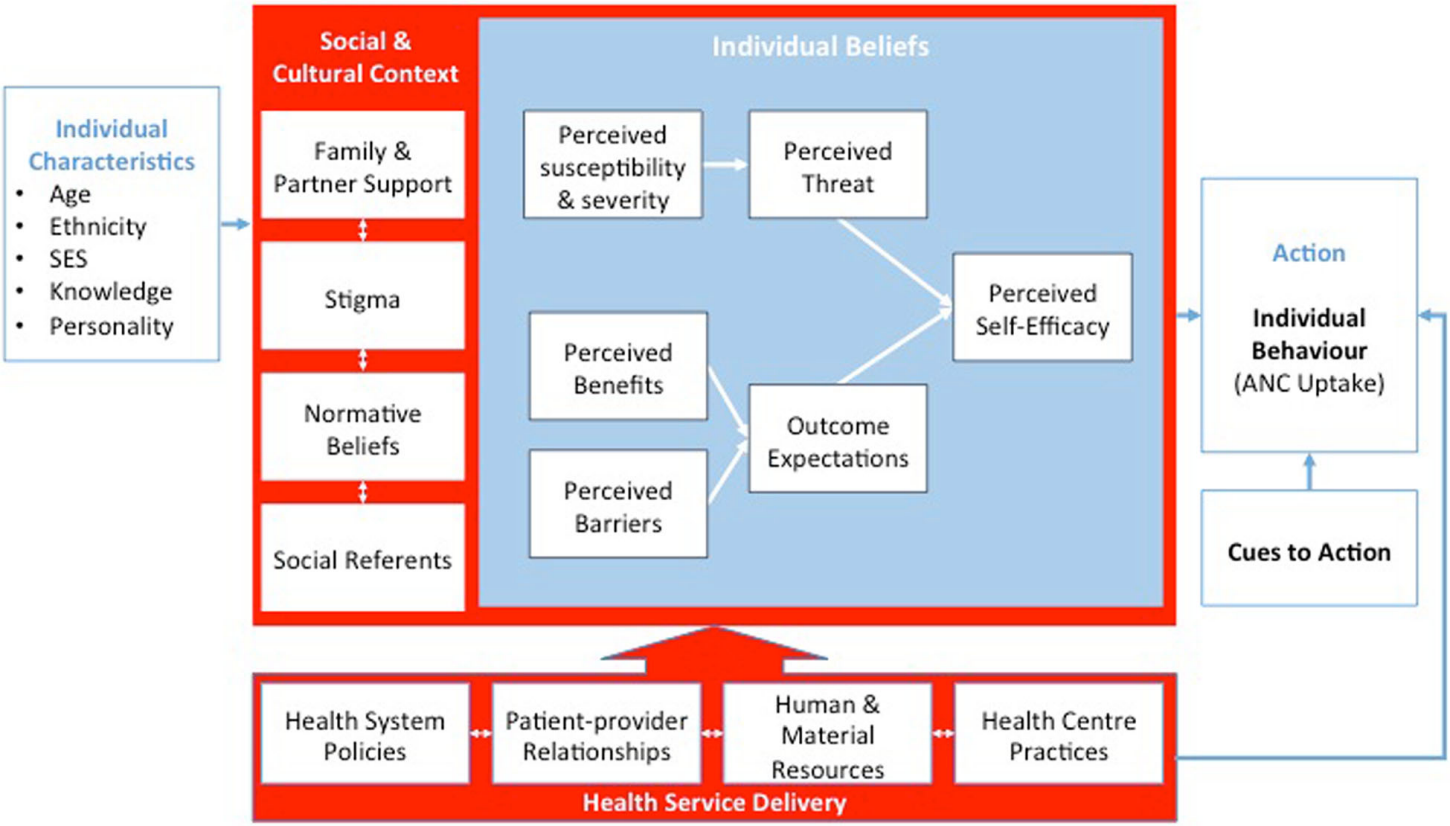
Adolescent ANC Model outlining the factors that motivate and facilitate ANC uptake among young mothers

Each individual-level construct shapes the adolescent’s belief in her ability to successfully utilize ANC (i.e her *self-efficacy)* (Fig. 2). Participants spoke about overcoming their shyness and fears in order to attend ANC and of weighing perceived benefits and barriers of attending. They expressed a belief that some people are just less “shy” by nature (or have higher self-efficacy to access health services).

### Social & Cultural Context

Participant narratives suggest that adolescent girls’ health-seeking decisions are highly influenced by sociocultural factors (summarized in Table 4). Many participants alluded to a strict cultural definition of what constitutes “proper” timing for motherhood and acknowledged that by this definition, they were too young to become mothers. The stigma associated with teenage pregnancy and early childbearing represented a powerful deterrent to attending ANC, and led to passivity among adolescents when they did attend:

*“When we were schooling, our mothers advised us not to take boyfriends, but because we were disobedient and took boyfriends we ended up getting pregnant. So when we go to the hospital, we are shy.” [GHA-5, R8]*

**Table 4:** Social & Cultural factors influencing ANC uptake among adolescents

Construct	Definition and application to ANC uptake by adolescents	Illustrative Quote(s)
Family and social support	The degree of involvement of other household members and parties associated with the pregnancy. ANC attendance is more likely in situations where there is a male ‘taking responsibility’ (financially) for the pregnancy or where there is support from family members. This includes financial and psychosocial support.	*“If she has someone who supports her then she will be able to go, if not she cannot go. There are some who are unable to go because when they got pregnant they were sacked from home by their parents. So when it happens like that she has no helper who will support her financially to attend ANC.” [GHA-2, R6]* *“It was my partner who gave me money to begin attending antenatal.” [GHA-2, R4]* *“Sometimes too your husband does not have money to give you and you have no other place to get money from. You will have to wait for him to get money from wherever he can get if from.” [GHA-5, R10]* *“I was living with a sister of mine who has delivered two children. So even when my mother mentioned it, I said I was afraid and all that. She was the one who advised me that they were good and they will give me drugs for me to deliver safely” [GHA-1, R1]*
Social referents	People in the individual’s life with the power to influence the individuals’ perceptions of a behaviour. ANC uptake is more likely when the individual has social referents who believe ANC is important. Types described by participants: 1. Formal authority figures 2. Role models with relevant lived experience	*“It is because she [her mother] is the one who gave birth to me and knows what happens during pregnancy so that is why if she tells me to go, I would go.” [GHA-2, R2]* *“Our fathers have never delivered a child before and they know nothing about pregnancy; that is why if they tell us to go we are less likely to go.” [GHA-2, R1]*
Normative Beliefs (Social norms)	An individual’s perception of pressures to perform (or not to perform) a behaviour based on prevailing ideas, culture and value systems. ANC uptake is more likely where normative beliefs encourage ANC attendance and are tolerant of adolescent pregnancy. Participants described prevailing beliefs/norms which impacted ANC use: • Best source of care (western vs. traditional medicine) • Acceptable age for motherhood • Who ‘ought’ to attend ANC • Stereotypes of nurses’ personalities	*“During the older days there was no hospital so our mothers never went for antenatal. But now with modernization there are hospitals, so those who refuse to go are still living in the past” [GHA-3, R3]* *“For some people they take local medicine at home. So they know that even if they do not go to the hospital, because they take local medicine from home they will give birth [at home]” [GHA-1, R6]* *“[Our peers] say it is stupid that we are going for antenatal. Because the new crop of nurses they are young so if you go there and you are young they insult you so they tell us not to go but take medicine from home.” [GHA-1, R6]* *“Some of the girls also say that they’re already pregnant and they only must be sure their baby kicks in the stomach. And so God will take care of them until they deliver. They also have the notion that even if they go for those sessions, the midwives only give them lots of drugs which do not help them in any way.” [GHA-6, R3]* *“We are females, but there is an age you must first attain. When we were going to school we were taught the age we must attain first before we get pregnant but for us we didn’t attain that age… that is why we are shy.” [GHA-5, R5]*
Stigma	Negative stereotypes perpetuated by individuals’ opinions and behaviours. ANC uptake is more likely where there is lower stigmatization of adolescent pregnancy (or where there are positive social referents challenging the prevailing stigma on behalf of the pregnant adolescents). As described by participants, main sources of stigma arise from: 1. Peers and social circle 2. Health care providers	*"Sometimes, if we go for the test and it is confirmed that we are pregnant and we go back home and tell our friends, when we are passing by our friends will be murmuring saying, “look at this girl she is not grown and she is pregnant and look at what it has done to her.” [GHA-5, R4]* *“Sometimes too they [healthcare providers] complain, saying that we are not grown and have gone following after boys then we get pregnant we come and bother them at the hospital” [GHA-5, R4]* *“Because the elderly are around [during ANC clinics] they keep on staring at you and then you begin to feel embarrassed” [GHA-4, R3]* *“Some of the nurses are rude and ill-mannered. There is one at [the clinic], because of her I don’t like going there, they look down on you and they think [more] highly of themselves than others.” [GHA-3, R4]*

In both countries, service provider attitudes played an important role in reinforcing adolescents’ ‘normative beliefs’ regarding the most socio-culturally acceptable time to get pregnant.

Positive social referents are individuals with the ability to influence the individual’s likelihood of acting to help themselves. Participants described two main types of social referents: a) formal authority figures and b) role models with relevant experience. Formal authority figures included fathers, male partners and health care providers (particularly nurses) – if these individuals believed ANC was important, it had an impact on the participants’ beliefs. However, participants placed the most emphasis on the opinions of those who had gone through childbirth themselves. If a mother, sister or female in-law had a positive prior ANC experience, then adolescents were reportedly more likely to attend.

### Health service delivery

In addition to individual-level and sociocultural factors, adolescent participants discussed several health systems-level factors that influence ANC uptake. These issues are not under the control of the individual yet have a marked impact on her ANC experience, including ease of accessing care as well as the quality of the services received. As summarized in Table 5, four themes emerged under this level: health system policies; patient-provider relationships; human and material resources; and health centre practices.

**Table 5:** Health service delivery factors influencing ANC uptake among adolescents

Construct	Definition and application to ANC uptake by adolescents	Illustrative Quote(s)
Health Systems Policies	National, or high-level, rules dictating patients’ access to health services. ANC uptake is more likely where policies do not discriminate against adolescents. Problematic policies: • Clinics requiring women (including adolescents) to bring their partner to ANC visits • Pregnancy not permitted at school (Tanzania only)	*“I disliked that we were told that we must bring men who got us pregnant. I disliked it because sometimes you are pregnant but the man is no longer in the picture. But still they tell you that they won’t attend you until you bring the man. Where will I get him?” [TZA-3, R3]* *“I was late [in seeking ANC] because I was scared that the clinic will tell me to go with my husband.” [TZA-3, R1]* *M: “Any other reason why young women are late to start attending ANC? R3: Sometimes it is when the woman is a student” [TZA-5]*
Patient-provider relationships	Interactions between individual patients and their health care provider at point-of-care. Participants described three categories of relationships: 1) Motivating and inspiring interactions with health care providers 2) Neutral, purely transactional interactions Inhibitory and discriminatory interactions. ANC uptake is more likely when participants developed meaningful, supportive relationships with health care providers. Positive interactions were particularly important for motivating repeat ANC visits.	*“Sometimes [the health care providers] get so angry and rude. They rip your clinic card into pieces” [TZA-3, R4]* *“I was afraid that, if I went, she (the nurse) will tell me that I was going to school and had not completed but have got myself pregnant, she will insult me. But when I went she held my hand and took me to the madam who attended to me really well.” [GHA-1, R1]* *“My sister-in-law said the nurses treated her harshly and rudely, so if I go they will treat me the same, but fortunately for me I experienced none of that so the nurses should treat us well.” [GHA-3, R10]* *“Madam sometimes even when you are pregnant and you go there and you are shy, you are not happy and you can frown. But if the nurse likes you she can say something funny for both of you to laugh at to create happiness.” [GHA-5, R4]* *“When a woman is dirty, they took a long time waiting to decide to attend you.” [TZA-6, R5]*
Human and Material Resources	The resources (human and material) necessary for implementing ANC at the health centre. ANC uptake is more likely when participants know that services and medications will be consistently available. Key issues described by participants: • Staff shortages • Medication stock-out • Lack of obstetric equipment and other materials (ANC cards)	*There is no water supply at the facility. When you go there to deliver you need to bring a bucket of water with you. [TZA-4, R4]* *“I disliked the waiting time. You go there and wait for many hours and the nurse says they are going for tea break.” [TZA-8, R1]* *“They did everything for me but there is only one midwife so we have to be delivered by the traditional birth attendant” [GHA-4, R2]* *“When you arrive at the clinic they tell you that the [ANC] cards are finished and that you should buy a notebook. Then they use that notebook to record all your details until you give birth.” [TZA-3, R1]* *“Some of the nurses would also give you prescriptions to go and buy certain drugs when you don’t have enough blood [have low iron in th blood], meanwhile they have the drugs which they can give to us and we also have no money to buy them.” [GHA-3, R7]* *“Sometimes, even if you have paid for the community health insurance (CHF), when you go to the facility they tell you to go outside to buy medicine, so the CHF is useless.” [TZA-3, R5]*
Health Centre Practices	The operationalization of health systems policies, as well as specific approaches to scheduling and delivering services; interpretation of policies and rules may vary from one healthcare provider to the next. ANC uptake is more likely when frontline workers are interpreting policies and practicing in a non-discriminatory manner, and when health centres are known to operate smoothly. Key problematic practices described by participants: • Partner testing for STIs (mainly HIV) at first visit (Tanzania only) • Illegal fees and inconsistent provision of medications • Opaque or unfair scheduling and triaging practices • Problems with referrals and transfers • Only conducting ANC clinics on specific days rather than on all days. • Lack of privacy and confidentiality for adolescents	*“When I went for ANC she asked me to sit down and I waited. But later I realized that she was attending to people who came to meet me while I was still sitting. Do you think it is good? So I dislike her a lot.” [GHA-3, R4]* *“I remember that if you are new there and you are being given a card, you will not be given a drug until you have done labs and taken the scan. So no matter how many times you come there they won’t give you the drug unless you go to [The referral hospital].” [GHA-1, R8]* *Sometimes we have to be transferred to [a larger facility] where we have no money for the service" [Gha-4, R2]*

In Ghana, some participants did not consider ANC to be adolescent “friendly” and believed that more could be done to tailor services to their needs. Attitudes of health care providers, or adolescents' anticipation of provider attitudes towards them, were particularly salient in participants' reflections. Participants often felt stigmatized by service providers for being school-aged and pregnant, and several participants noted feeling ostracized by older women attending ANC sessions. These experiences shaped their belief that ANC was not intended for them. One participant expressed:

*Actually, at the age when you are supposed to give birth, we have not attained it yet…. So when I see that I am the only young girl there and not being attended to immediately, that is what will make me think and ask myself, “Ah! Why is it that the adults are being attended to and I am not?” I can ask that and someone will tell me that [ANC] is for adults and not for children" [GHA-5, R4]*

In contrast, a majority of participants in Tanzania felt services for pregnant women were comparable, regardless of age, and that ANC was equally suited to adolescent girls and older women. In one FGD, two participants felt that adolescents actually received higher quality services compared to older women “*because it is the first time to have a baby. The services and education are of higher quality than that of experienced women*” [TZA-4, R3].

### Adapting the health belief model to explain ANC uptake among adolescents

In the adapted HBM (Fig. 2) we posit that an individual adolescent mother is defined by a set of underlying personal characteristics (white box on the left), and this individual engages in a decision-making process about a health-related behaviour – in this case, whether to access ANC. The decision-making process is dependent on individual-level decision-making, (represented by the blue box), but is also embedded within a set of household- and community-level variables (surrounding red box). The latter set of variables was added based on our review of existing literature and emergent findings in the present study. We also added the health service delivery component to the model (red box at the bottom) to represent constructs from ecological models of health service utilization. These variables correspond to the most distal health systems and policy-level factors, which are largely out of individuals’ control yet indirectly influence adolescents’ uptake of ANC.

## Discussion

This study is the first to develop an adapted theory-based framework illustrating the complexity of adolescent care-seeking during pregnancy in two sub-Saharan African settings. Drawing on constructs of the HBM, we demonstrate that while an adolescent mother is capable of exercising her own agency and may be viewed as a rational decision-maker, she is also developmentally vulnerable to external influences and must be supported in her ability to make autonomous decisions. Interpreting results through an adapted HBM demonstrates that adolescent health-seeking behaviour can vary widely among individuals and within communities, is shaped by the opinions of family members and peers, and is intrinsically influenced by broader health systems-level factors. While the model presented here focuses specifically on ANC utilization, it may also be used to understand how adolescents engage with health services more broadly.

Many of the factors that influence adolescents’ ability to access ANC lie beyond the health facility itself, and overlap with those documented for older women in LMIC [9, 22]. However, findings suggest that adolescents grapple with additional negative individual and societal responses to early childbearing, and may therefore have less freedom to access services compared to older mothers. For example, lack of financial and decision-making autonomy [9] and challenges related to distance and transport [23] are widely acknowledged as important determinants of ANC utilization, but may be more salient for adolescents due to their social positioning. Previous research in Tanzania reported that women who had unplanned pregnancies tend to be more hesitant to attend ANC [24]; this resonates with the experiences of many adolescents in our study, who expressed embarrassment due to unexpected early pregnancy and consequently, reluctance to attend ANC.

To ensure that ANC is accessible to adolescents, it is essential to address their particular needs and preferences. Recognizing that health services for adolescents are often fragmented, poorly coordinated and of inconsistent quality, WHO recently published new *Global Standards for Quality Health-care Services for Adolescents* [25]. While the present study was not designed with WHO standards in mind, it is relevant to note that participant narratives resonate strongly with, and add credence to, several of these recommendations. In particular, adolescents highlighted the critical role of community support, providers’ competencies, facility characteristics (e.g. maintenance of privacy/confidentiality), and equity and non-discrimination in facilitating ANC access. We did not assess ‘adolescent friendliness’ of health facilities in this study; future research to compare adolescent perceptions of service quality with implementation quality of the WHO standards in target facilities is warranted.

The WHO standards outline a number of concrete actions to be taken at facility level to improve care for adolescents without overly burdening health care providers. While these standards are specific to youth, they educe a standard of care that should be met for *all* individuals. Previous research in South Africa found that adolescent mothers prioritized interactions with health care providers, wait times, comfort level of the facility, quality of health education and support received for childbirth and parenting as factors determining ANC use [26]. Similarly, we found that service provider attitudes and the strength of patient-provider relationships are a major determinant of adolescents’ willingness to attend ANC in Tanzania and Ghana. Narratives suggest that positive interactions with ANC providers may be the most important determinant of perceived quality of care – even more so than the content of care itself. These findings align with recent efforts to mainstream respectful maternity care for women of all ages along the continuum of care [27, 28].

The two sites for this study are culturally and geographically distinct, and qualitative findings were drawn from a small number of participants. Findings may therefore not be generalizable to adolescents external to our study. A higher proportion of Tanzanian participants were married, which may partially explain differences in findings between countries, however this study was not designed to make such comparisons. The observation that participants in both Tanzania and Ghana identified similar motivators and facilitators of ANC uptake and valued similar aspects of service provision is important. Furthermore, these themes are consistent with concepts previously identified in studies of the experience of adolescents engaging in ANC in other sub-Saharan African countries [11, 29, 30]. These commonalities illuminate critical targets for policy and programs that could have a marked impact on adolescents’ uptake of prenatal health services in resource-constrained settings.

A notable limitation of this study is that six participants had delivered more than 12 months prior to data collection, which may have introduced recall bias. Furthermore, focus group discussions in Ghana were more rich and nuanced than discussions in Tanzania, despite comparable training of facilitators in each site. This may reflect differences in interviewing skills between facilitators or differences in participants’ willingness to discuss their experiences. Future studies with adolescent mothers in Tanzania may consider complementing focus group discussions with one-on-one in-depth interviews to address this possibility. Despite these limitations, this study was strengthened by a rigorous approach to quality assurance that included regular debrief meetings throughout data collection, and systematic review of each transcript for accuracy and consistency.

Future research must build upon these findings by continuing to explore the experiences and preferences of adolescents, including those who have *not* accessed ANC, and by investigating the relevance of the adolescent-oriented Health Belief Model in other settings. Additionally, developing and testing interventions that address both individual-level determinants of adolescent health-seeking, as well as underlying sociocultural determinants, including gender inequality, should be a focus of future studies.

## Conclusions

Consistent with the WHO’s new “recommendations on antenatal care for a positive pregnancy experience”, our findings add a more nuanced examination of adolescents’ experiences with health services, and are intended to guide discussions around adolescent friendly health policies and programs in settings where health and human resources are constrained. The development and use of an adapted theory-based framework facilitated our demonstration of the complexity of adolescent care seeking during pregnancy in resource-poor settings.

## Additional files

Additional file 1: ANC Study: Focus Group Discussion Guide. (PDF 354 kb)

Additional file 2: Transcription and translation: Reviewer Assessment Form. (PDF 632 kb)

## Abbreviations

ANC: Antenatal care
FGD: Focus Group Discussion
HBM: Health Belief Model
HIV: Human immunodeficiency virus
LMIC: Low and middle-income countries
NGO: Non-governmental Organization
SD: Standard Deviation
STI: Sexually transmitted infections
WHO: World Health Organization
