# Supplementary material for: How can engagement of adolescents in antenatal care be enhanced? Learning from the perspectives of young mothers in Ghana and Tanzania
Source: BMC Pregnancy Childbirth. 2019 May 23;19:184. doi: 10.1186/s12884-019-2326-3 (PMC6533671; doi:10.1186/s12884-019-2326-3)
Supplement: Supplementary file 1 — ANC Study: Focus Group Discussion Guide. (PDF 354 kb) [file 12884_2019_2326_MOESM1_ESM.pdf]

ANC Study: RA's Focus Group Discussion Guide

Introduction [RA to read aloud to the whole group]

Thank you all very much for joining me today. My name is [insert RA name] and I will be leading today's discussion about antenatal care. I have a colleague with me called [insert name of note taker] who will assisting me and writing down notes.

We are working with a research team from Canada who are conducting a study in Ghana and Tanzania to learn about the experience of young mothers who have used antenatal care services during their pregnancy, and to investigate how these services could be made more accessible to young women. In the end, the goal is to contribute to improving the health of mothers and children by providing recommendations to the organizations who provide ANC services.

You were asked to participate in this study because we are interested in hearing about your experience and ideas, as a young mother who went to some antenatal care visits during your pregnancy.

Your safety is very important to us, and we want you to feel comfortable sharing your opinion. As I explained earlier, your comments will be kept confidential by the research team, so your name or other identifying information will never be used. I also ask that you do not share the things that your peers have said today when you return to your homes.

You are free to participate as little or as much as you want in the discussions. And you are free to leave at any point, if you wish. As I told you before, we will be recording the discussion so that the research team will have very detailed information about your ideas.

If you have any questions during the discussion, please ask me. Before we get started, my last request is for everyone to be respectful of their peers. This means that when someone else is talking, we allow them to finish and that we do not use hurtful or insulting language/behaviour towards other participants. We all have our own opinions and should feel free to express them, and to discuss in a safe environment.

This discussion will take about two hours in total, and we will have a break partway through so we can all have some refreshments.

Icebreaker Activity:

As per your training and orientation with the study team, select 1-2 icebreaker activities so that the participants can introduce themselves and have the opportunity to become more comfortable.

Now you can begin the discussion, following the guide below:

Section/Question	Question and activity guide	Notes (RA may quickly jot notes as reminders; the Note Taker must write detailed notes throughout)
A. Initial questions: Experience of ANC services		
A.1	What are your general impressions of ANC services?	

A.2	What do you remember about your experience attending ANC sessions?	
A.3	Starting with the first ANC session you attended, can you tell me about what happened?<br><br>***Activity:*** *Start with the first visit, and walk through the process with the group. Use the chart paper to write down each step of the first ANC visit. Once the steps have been written down, revisit each step and probe:*<br><br>What happened specifically at this stage? How did that make you feel?<br><br>What were your impressions of the ANC service at this stage?	
A.4	How was your experience at subsequent ANC visits, if you attended more than one? In what ways did your experience differ from the first session? In what ways was it the same?	
A.5	What did you like the most about the ANC sessions?<br><br>*Probe for additional responses. Use prompts like:*<br>*Can you tell me more about this?*<br>*What else did you like?*<br>*Does anyone else have a similar or a different idea to add?*<br>*Is there anything else you would like to share?*	
A.6	What was missing from the ANC sessions?<br><br>*Probe for additional responses. Use prompts like:*<br>*Can you tell me more about this?*<br>*What else did you like?*<br>*Does anyone else have a similar or a different idea to add?*<br>*Is there anything else you would like to share?*	
B. Awareness of ANC services		
B.1	How did you come to know about ANC services?<br><br>*Prompt for more individuals to share their ideas:*<br>*Did anyone else find out about ANC services in this way?*<br>*What other ways did you hear about ANC services?*	
B.2	How would other young girls in your community find out about ANC services if they became pregnant?<br><br>*Write these down on the chart paper*	
B.3	***Activity****: Read through the full list of ways that girls might find out about ANC services and ask the participants to rank the list from most important to least important (#1, 2, 3 etc).*	

	If participants disagree, ask them to explain their reasons. Give participants time to explore their different opinions, and then ask them to come to an agreement collectively.	
B.4	Who might not know about ANC services in your community?	
B.5	How did you come to know about ANC services?<br><br>*Prompt for more individuals to share their ideas:*<br>*Did anyone else find out about ANC services in this way?*<br>*What other ways did you hear about ANC services?*	
C. Timing of ANC		
C.1	***Activity**: Draw a long horizontal line on the chart paper, and divide this line into 10 months using small vertical lines. Explain to the participants that this line shows a pregnancy, from conception (the far left) to birth (the far right).*<br><br>*Ask the participants to tell you where to mark down the timing of each ANC visit that young mothers would go to.*<br><br>*If there is disagreement between participants, probe into what makes them disagree.*<br><br>*Remind participants that this is not about what you are "supposed" to do, it is about what young mother actually do.*<br><br>*Once the different opinions have been thoroughly explored, ask the group to come to an agreement about a 'typical' young pregnant girl in their community.*<br><br>When does this girl attend ANC visits? How many does she attend?<br><br>*Continue the discussion until the participants come to agreement, and mark the timing of each visit down on the timeline.*<br><br>*Then ask probing questions about the reasons behind the number of visits and the timing of the visits.*<br>(For example, if the first visit is late in pregnancy, ask what are the reasons that a girl might attend her first ANC visit late in her pregnancy?	
D. Facilitators and barriers		

D.1	*Ask the participants to imagine a 'typical' young pregnant girl from their community. [You could make up an example to describe a fictitious girl who is similar to the girls in the FGD].*<br><br>*Tell the participants that this young girl was able to attend 4 ANC sessions, and that her first session was early in her pregnancy. Then ask:*<br><br>What are the reasons that this girl was able to attend her 4 ANC sessions?<br><br>*Probe:*<br>*Any other reasons?*<br>*What things helped her to be able to attend? (This could include people, knowledge, beliefs, services, etc.)*	
	Now ask the participants to think about another 'typical' young pregnant girl from their community. [You could make up another example to describe a fictitious girl who is similar to the girls in the FGD]<br><br>*Tell the participants that this girl was not able to go to ANC sessions. Then ask:*<br><br>What are the reasons that this girl was not able to attend her 4 ANC sessions?<br><br>*Probe:*<br>*Any other reasons?*<br>*What things stopped her from being to be able to attend? (This could include people, knowledge, beliefs, services, etc.)*	
D.2	*Tell the participants that most women in Ghana are somewhere in the middle, between these two examples. Most women are able to attend one or two visits. But it is often later on in their pregnancy.*<br><br>*Revisit the facilitators and barriers to attending ANC (that emerged during the previous two questions), to ask for more specific information on the themes that were mentioned:*<br><br>What does [insert theme/idea] mean specifically? Can anyone provide an example?<br><br>How often does [insert theme/idea] happen?<br>In your community, who experiences [insert theme/idea]?<br>Why do you think individuals might experience [insert theme/idea]?	

	How might [insert theme/idea] change throughout the course of the pregnancy? (Or do they remain the same always?)	
E. Importance of ANC		
E.1	What, if any, are the reasons that a young woman should attend ANC sessions during pregnancy?<br><br>*Probe: Why do you think that is a reason?*<br>*Any other reasons?*	
E.2	What, if any, are the reasons that a young woman should not attend ANC sessions during pregnancy?<br><br>*Probe: Why do you think that is a reason?*<br>*Any other reasons?*	
F. Improving ANC services		
F.1	What are some ways that ANC services could be improved to be more friendly towards young women?<br><br>*Tie the conversation back to any ideas mentioned in earlier questions.*<br><br>*Probe:*<br>Any other ideas?<br>How could we motivate more young women to use ANC services when they are pregnant?<br>What would be the most important improvement to make?<br>Who should make these improvements?	
F.2	Is there anything else you have thought of during today's discussion that you would like to talk about? We can revisit a previous question, or you can mention a completely new idea.	

Wrap-up and thank you:

- Thank all of the participants for their contributions to the discussion
- Remind the participants that the information shared in today's discussion is meant to be confidential. So they should not share the information with other people who were not part of the FGD.
- Remind participants that when this study is completed, they will be able to access the results from the local NGO office (provide contact details if needed).
- Distribute the token of appreciation (if applicable)
- Address any needs for transportation reimbursement (if applicable)

Participant Characteristics Questionnaire

Research assistant instructions:

This survey is to be administered by the research assistant, as the participants arrive, after obtaining informed consent and prior to the start of the focus group discussion.

Introduce yourself and ask the participant if they are willing to answer a few short questions about themselves. These questions will help the research team to understand what types of young mothers were present during the discussion. Answers will not be shared with any other participants, and names will not be written down on the questionnaire (the answers will be anonymous).

**Question number**	**Question**	**Answer options**
1.0	What is your age? (in complete years)<br><br>*Read question aloud*	*Please write age (years)*<br><br>_____
1.1	What is the age of your child? (in complete months)<br><br>*Read question aloud*	*Please write age (months)*<br><br>_____
1.2	What is your current marital status?<br><br>*Read question aloud & then answer choices aloud*	*Please select one answer choice:*<br>1. Married<br>2. Common law<br>3. Single<br>4. Divorced/separated<br>5. Other. Specify: _________________
1.3	Where did you attend your ANC visits?<br><br>*Read question aloud*	*Please write the name of the health facility:*<br><br>_____________________________
1.4	How many ANC visits did you attend while you were pregnant?<br><br>*Read question aloud.*	*Please enter the number of visits:*<br>_____
1.5	When did you attend your first ANC session?	*Please enter the month of pregnancy (gestation). If timing of first visit is not known, write "unknown".*<br><br>_____________________________

1.6	Are you currently pregnant?<br><br>*Read question aloud & then answer choices aloud*	*Please select one answer choice:*<br>1. Yes<br>2. No<br>3. Did not respond
1.7	*If yes to question 1.6:*<br><br>Are you attending ANC sessions?<br><br>*Read question aloud & then answer choices aloud*	*Please select one answer choice:*<br>1. Yes<br>2. No<br>3. Did not respond
1.8	I*f no to question 1.8*:<br><br>If you were to become pregnant again, do you think you would attend ANC sessions?<br><br>*Read question aloud & then answer choices aloud*	*Please select one answer choice:*<br>1. Yes<br>2. No<br>3. Did not respond
