# Supplementary material for: How can engagement of adolescents in antenatal care be enhanced? Learning from the perspectives of young mothers in Ghana and Tanzania
Source: BMC Pregnancy Childbirth. 2019 May 23;19:184. doi: 10.1186/s12884-019-2326-3 (PMC6533671; doi:10.1186/s12884-019-2326-3)
Supplement: Supplementary file 2 — Transcription and translation: Reviewer Assessment Form. (PDF 632 kb) [file 12884_2019_2326_MOESM2_ESM.pdf]

Perceptions and experiences of adolescent mothers accessing antenatal care services in Volta and Eastern Regions, Ghana, and Singida and Iramba Regions, Tanzania

Transcription and translation: Reviewer Assessment Form

Study Background
We have conducted focus group discussions (FGDs) in Eastern and Volta regions of Ghana, with adolescent mothers who have delivered only once, and used antenatal care (ANC) services during their pregnancy. We recruited participants from 4 different health facilities and conducted 6 FGDs in total. We focused on Health Centres that have been supported through the WATCH Program (run by Plan Ghana with the support of Plan Canada).

The research team at SickKids developed the study protocol and will be conducting the final analysis, with input from our NGO partners. Plan supported the research team in selecting the Health Centres and inviting the participants. However, the FGDs were conducted independently in a neutral location (neither Plan nor the Health Centre staff were involved in the FGDs). We hired two independent Research Assistants who spoke the local languages (Twi, Ewe and Krobo). The Research Assistants conducted the FGDs and also translated and transcribed the audio recordings.

The Research Assistants used a simultaneous translation and transcription process: they listened to the audio recording in the original language and transcribed the conversations in English.

Purpose of the Independent Checks
We want to make sure that the transcripts capture the whole story of what the participants were trying to communicate. This is important in order to conduct a high quality analysis and to draw conclusions that are meaningful and helpful to all the stakeholders (community, non-governmental organizations and funding partners).

Our Research Assistants worked very hard to conduct the FGDs and put a lot of time into translation/transcription. But since translation can be somewhat subjective, and since the RAs were immersed in the study, we are asking you to complete an independent check step in order to provide a second, non-biased opinion. This will help reduce the chances of accidental errors, misinterpretations, or omissions etc.

The transcripts should be thorough and in-depth, and should be a written record of the entire FGD conversation. There should be no summarizing or skipping parts of

the conversation. We will be conducting a detailed thematic analysis, so it is important to have as much "raw data" as possible in the transcripts. A good, complete transcript should include indications of any pauses, laughter, repeated questions, and any "filler" words (e.g. um, uh, ahh, etc.). A good transcript might also contain comments in square brackets [these comments provide additional information to the reader to clarify anything that might be confusing for people who were not present during the discussion].

Please keep in mind that a high quality translation is not necessarily "word-for-word" because certain expressions/idioms may be different between the original language (Ewe, Twi or Krobo) and the final language (English). The important thing is that the original, intended meaning is the same.

Instructions

1. You have been provided with transcripts and audio files for each FGD that we would like you to review. Each audio file is named the same as the matching transcript - please ensure that you read the transcript that corresponds to the audio file from the same FGD.
2. Please conduct a separate assessment for each FGD that you have been assigned. (You will complete this document separately, for each FGD that you were assigned. For example, if you are checking 4 FGDs, you will complete 4 separate assessment forms.
3. Follow the steps in the tables below.
4. Email Lindsey at [redacted] if you have any questions or concerns, or call on Skype.
5. Send this completed document, plus the edited transcript (according to the instructions below) back to Lindsey by the arranged deadline.

**Reviewer's Checks and Comments**	
Focus Group Discussion ID<br>Include the location name and #1 or #2, if applicable<br>(For example: Adawso #1)	
A. Personal details	
Reviewer's name:	
Reviewer's institution and title:	
Date of review:	
B. General impressions of the transcript	
Instructions: Please listen to the first 10 minutes of the audio recording and read along with the transcript. On the transcript, please use the highlight tool in Adobe to mark portions of the text that you think have problems. Then use the comments tool in Adobe to make any additions, corrections or deletions that you think are needed to improve the quality. In the box below, please describe your overall impression of the translation & list any specific concerns. Please refer to any examples that may help to justify your impressions.	

C. Additional checks
Instructions: Please listen to the audio recording at the specific time-points listed below. Read the English transcript as you listen to the audio recording segment (time points and page numbers are listed below). On the transcript, please use "Track Changes" to make any additions, corrections or deletions that you think are needed to improve the quality.<br>In the boxes below, please describe your overall impression of the translation & list any specific concerns (in 2-5 sentences).

Last 10 minutes of the FGD	
One random spot in the middle (please indicate the time point)	

**Reviewer's Assessment**		
Please provide a numerical score from 1-5 for each of the categories listed below. Then provide an overall score, and any additional comments.<br>1 = Needs major revisions<br>3 = Needs some revisions, but the issues are minor<br>5 = No revisions needed		
Category	Description of the Category	Grade Assigned (1 to 5)
Completeness of transcript?	Do all of the questions and answers that you heard on the audio file appear in the English transcript? (In other words, there is no skipping or condensing parts of the conversation)	
Reliability of translation?	Overall, is the meaning of the original questions and answers captured fully and accurately in the English version?	
Consistency and accuracy of terminology?	Are the English terms/words that were used the best possible translation of the Ewe/Twi/Krobo words? Is there consistency throughout the transcript in the way that specific words are translated?	
Overall impressions of quality?	Considering the three categories listed above, and your overall impressions, what is your assessment of the quality of the translation?	
Any additional comments or concerns?		
